# Species sensitivity assessment of five Atlantic scleractinian coral species to 1-methylnaphthalene

**DOI:** 10.1038/s41598-020-80055-0

**Published:** 2021-01-12

**Authors:** D. Abigail Renegar, Nicholas R. Turner

**Affiliations:** Nova Southeastern University, Halmos College of Natural Sciences and Oceanography, Dania, FL USA

**Keywords:** Marine biology, Environmental impact

## Abstract

Coral reefs are keystone coastal ecosystems that are at risk of exposure to petroleum from a range of sources, and are one of the highest valued natural resources for protection in Net Environmental Benefit Analysis (NEBA) in oil spill response. Previous research evaluating dissolved hydrocarbon impacts to corals reflected no clear characterization of sensitivity, representing an important knowledge gap in oil spill preparedness related to the potential impact of oil spills to the coral animal and its photosymbiont zooxanthellae. This research addresses this gap, using a standardized toxicity protocol to evaluate effects of a dissolved reference hydrocarbon on scleractinian corals. The relative sensitivity of five Atlantic scleractinian coral species to hydrocarbon exposure was assessed with 48-h assays using the reference polycyclic aromatic hydrocarbon 1-methylnaphthalene, based on physical coral condition, mortality, and photosynthetic efficiency. The threatened staghorn coral *Acropora cervicornis* was found to be the most sensitive to 1-methylnaphthalene exposure. Overall, the acute and subacute endpoints indicated that the tested coral species were comparatively more resilient to hydrocarbon exposure than other marine species. These results provide a framework for the prediction of oil spill impacts and impact thresholds on the coral animal and related habitats, essential for informing oil spill response in coastal tropical environments.

## Introduction

Coral reefs are one of the world’s most threatened ecological resources due to a variety of environmental stressors, and have an elevated risk of exposure to petroleum hydrocarbons due to their proximity to the coastline. During the 2010 Macondo incident in the Gulf of Mexico, tides, currents, and winds moved oil and dispersed oil near sensitive resources, but fortunately did not impact shallow-water coral reefs. However, coral reefs of the US and elsewhere are ecosystems that have been, and could be again impacted by future spills and mitigation measures^1–3^. Detailed knowledge about exposure thresholds and impact pathways on coral reefs and tropical hard-ground communities is therefore needed to provide data for existing/emerging response support tools, and to allow the development of damage forecast scenarios as a tool for improved planning and decision-making. Previous research to evaluate hydrocarbon toxicity to corals and coral reefs has generally focused on community level effects^4,5^, and results are often not comparable between studies due to variability in bioassay methodology and toxicant utilized^6–8^. Thus, a significant data gap exists on the toxicity thresholds of hydrocarbons to corals.

Crude oil and its derivatives (e.g. petroleum products such as diesels and heavy fuel oils) are complex mixtures containing thousands of compounds, with significant variability in composition between different oils depending upon the source and manufacturing conditions^9^. For this reason, results of bioassays completed with one oil may not be relevant to those completed with another. Differences in toxicant preparation methods prior to exposure can also lead to profound effects on the distribution of constituent hydrocarbons in the test media, compounding the difficulties associated with extrapolation of effects. The toxic mode of action of most hydrocarbons found in crude oil mixtures is non-polar narcosis, which has been determined to correlate well with the octanol–water partition coefficient (log *K*_ow_) across many organisms^10^. The target lipid model (TLM)^11^ uses a universal narcosis slope to describe this relationship, which allows the prediction of threshold concentrations (LC50) for multiple compounds following experimental determination of the threshold concentration for one hydrocarbon. The organism’s critical target lipid body burden (CTLBB) can then be used to estimate the LC50 of other constituents, and as constituent hydrocarbons in oil have additive effects, the toxic unit approach can be utilized to estimate the toxicity of complex mixtures of hydrocarbons^12,13^. Although laboratory assays utilizing single hydrocarbon exposures are not representative of real-world oil spill environmental conditions, this methodological approach is recommended to support calibration and validation of toxicity models which predict environmentally realistic exposures^14,15^.

This research is primarily intended to fill critical information gaps for oil spill response decision-makers. It was developed with input from collaborators in government and the response community in order to design study outputs that would integrate with fate and effect models to better inform response decision-makers on the potential impact of transported spilled oil or dispersed oil on coral reefs. Specifically, the availability of reproducible toxicity endpoints for multiple species of scleractinian corals would support modeling of the potential effects of transported concentrations of dispersed oil plumes at various distances from coral communities. This study therefore included a series of experiments to assess the acute and subacute toxicity of the reference polycyclic aromatic hydrocarbon (PAH) 1-methylnaphthalene (1-MN) to five ecologically relevant Atlantic shallow-water corals (*Acropora cervicornis*,* Solenastrea bournoni*,* Stephanocoenia intersepta*,* Siderastrea siderea*, and* Porites astreoides*). A standard toxicity testing protocol for adult scleractinian corals, which considers coral response using multiple metrics^16^, was used to allow cross-species comparisons and develop a more complete picture of hydrocarbon toxicity to scleractinian corals. The target species represent five distinct genera found in western Atlantic coral reef environments which have a range of resilience to environmental disturbance^17^, and may therefore represent a broad assessment of the sensitivity of this taxonomic group to petroleum hydrocarbons. These new acute and subacute toxicological endpoints can be used to evaluate the potential impacts of hydrocarbons on scleractinian corals relative to other coral reef organisms, and are thus an essential tool for informing oil spill response decision-making in coral reef environments.

## Methods

### Experimental organisms

Corals used in the exposures were collected from shallow waters (collection depth range 6–10 m) offshore of Broward County, Florida and transported to the laboratory. Colonies of *P. astreoides*, *S. siderea*, *S. intersepta* and *S. bournoni* (collected in mid-May 2018) were cut into 4 cm^2^ fragments within 1 wk of collection, and numbered according to colony using small underwater paper tags attached with cyanoacrylate gel glue. Branch tips (5 cm in length) were cut from multiple colonies of *A. cervicornis* (collected in mid-March 2018)*,* and attached with a minimal amount of cyanoacrylate gel glue to small numbered aragonite bases on the day of collection. Corals were acclimated to laboratory conditions and allowed to heal in a 1100 L indoor coral culture system for a minimum of 2–3 weeks before pre-exposure data collection began. Corals were not target fed in this system, but nutrients were supplemented with a commercial coral-specific amino acid solution (Brightwell Aquatics CoralAmino, 7 mL 3 times per week). Artificial seawater (prepared with reverse osmosis water and Tropic Marin Pro-Reef sea salt) was used; the system was maintained at 35 PSU and 26 °C, with artificial light provided by Radion XR30 Pro (Ecotech Marine) LED lights (12 h photoperiod, programmed sunrise and sunset, ultraviolet radiation removed from spectrum, max PAR 220 μmol m^−2^ s^−1^ for *A. cervicornis*, and 120 μmol m^−2^ s^−1^ for all other species). The same programmed lighting regime was used, depending upon species, throughout all experimental periods.

### 1-Methylnaphthalene exposures

Each of the 5 experiments (a separate exposure was conducted for each species) included a 2 week pre-exposure period (corals maintained in the laboratory system) to establish baseline coral health, a 48-h constant exposure, and a 4 week post-exposure period (corals returned to laboratory system) to assess recovery potential. The 48 h coral exposures to 1-MN were conducted using a continuous-flow recirculating passive dosing methodology (described in Renegar et al.^16^) which employs polydimethylsiloxane (PDMS) O-rings as a partition-controlled chemical reservoir. Briefly, this method uses 24 individual dosing systems; each independent system consists of a single 500 mL clear glass exposure chamber connected to a single 2 L glass dosing vessel by a multi-channel peristaltic pump with Viton tubing (flow rate = 5 mL min^−1^) . Six treatments were used, including a seawater control and 5 concentrations of 1-MN, with 4 replicate dosing systems per treatment. No O-ring or methanol (MeOH) controls were used as these factors were previously demonstrated to have no significant effect^16^. Treatments were randomly assigned to dosing systems. Target 1-MN concentrations were chosen based upon the results of a previous range-finding exposure^16^.

Before the start of the exposure period, PDMS O-rings (O-Rings West) were cleaned by rinsing in ethyl acetate (Fisher Scientific) (24 h), methanol (Fisher Scientific) (3 × in 24 h), and deionized water (3 × in 24 h), then dried at 110 °C for 1 h. Stock solutions of 1-MN (Acros Organics, 97%) in methanol were prepared using the equation:

$${C}_{MeOH}= \left[{K}_{MeOH-PDMS}+ \left[\frac{{V}_{PDMS,A}}{{V}_{MeOH}}\right]\right]\times \left[{K}_{PDMS-Water}+ \left[\frac{{V}_{Water}}{{V}_{PDMS,D}}\right]\right]\times {C}_{Target,}$$

where C_MeOH_ is the concentration of hydrocarbon added to methanol (mg L^−1^); C_Target_ is the target concentration in seawater (mg L^−1^); V_MeOH_ is the volume of the methanol dosing solution (mL); V_PDMS,A_ is the volume of PDMS O-ring acceptor in the methanol stock solution (mL); V_PDMS,D_ is the volume of PDMS O-ring donor in the aqueous test media (mL); V_Water_ is the volume of water in the recirculating flow-through system (mL); K_MeOH-PDMS_ is the partition coefficient of the hydrocarbon between methanol and PDMS; and K_PDMS-Water_ is the partition coefficient of hydrocarbon between PDMS and water (K_MeOH-PDMS_ = 2.818 from Knap et al.^18^, and K_PDMS-Water_ = 1621.810 from Bera, G. unpub. data).

The calculated mass of 1-MN required for each experimental concentration was dissolved in methanol and mixed for 1 h. Cleaned PDMS O-rings were added to the methanol stock solutions and allowed 72 h (on an orbital shaker) for adequate partitioning of hydrocarbon into the O-rings^19^. Prepared O-rings were then rinsed with seawater (3×) and transferred to randomly assigned dosing systems. Each dosing system was filled with seawater (2.5 L, 35 PSU), filtered to 1 µm (Polymicro), from the laboratory coral culture system. Dosing systems had < 10% headspace when filled and operational, to limit volatile loss, and were vigorously stirred throughout. The peristaltic pumps were started and the systems were allowed 20–24 h for equilibration^19^.

After equilibration, corals were transferred to exposure chambers and the 48 h exposure was initiated. Three coral fragments were used in each treatment replicate; for *P. astreoides*, *S. siderea*, *S. intersepta,* and *S. bournoni*, coral fragments were from three different colonies, and each exposure chamber contained one randomly assigned fragment from each colony. Corals were not fed during the exposure, and lighting was provided as described above for the pre-exposure period. After the 48-h exposure, the exposure chambers were opened and surviving corals were transferred back to the laboratory system for monitoring during the post-exposure recovery period.

### Coral assessment

Percent recent mortality^20^ was used to visually estimate acute effects. Coral mortality was identified by severe tissue attenuation to the point of skeletal element exposure, or through sloughing of tissue after large amounts of swelling and mucus release. Partial coral fragment mortality also occurred in some species and was visually assigned a percent mortality score at 5% intervals. To estimate sub-acute effects, the visual condition of each coral was semi-quantitatively scored (including color, polyp extension/retraction, tissue swelling/distension, tissue attenuation, and mucus production). These individual characteristics, when taken together, represent a progressive physical expression of increasing sublethal stress that precedes partial tissue loss and/or complete mortality of the coral. Each of the five characteristics were scored on a scale of 0 (normal limits) to 3 (severely affected), with a precision level of 0.5 (thus half-scores are permitted). This scoring system is based in part on a histologically-verified stress index^21,22^, and has been adapted and expanded to reflect observed impacts of petroleum hydrocarbon exposure on the shallow-water corals used in these experiments. It has been previously applied to evaluation of hydrocarbon effects on the scleractinian coral *Porites divaricata*^16^, and is further described in the Supplemental information (Coral scoring matrix, Supplementary Table S1).

Photosynthetic efficiency measurements were used as an indicator of the physiological status of the autotrophic endosymbiotic zooxanthellae. The light adapted effective quantum yield [(F_m_′ − F′)/F_m_′ or ΔF/F_m_′] of the endosymbiotic zooxanthellae was determined from measurements of initial fluorescence (F′) and maximum fluorescence (F_m_′) by applying a saturation pulse of light using a pulse amplitude modulated fluorometer (Diving-PAM, Walz, Germany). A total of 4 measurements from living tissue were taken per coral (around the circumference of a branch tip, or 1 in each quadrant) to obtain a representative measurement for the coral fragment. For all measurements, the distance between the fiber optic cable (5.5 mm diameter) and live coral tissue was adjusted (2–10 mm) in order to maintain F′ readings between 250 and 400 units. Additional fluorometer settings used to determine ΔF/F_m_′ for each species are provided in Supplementary Table S2. To ensure that differences in photosynthetic efficiency were not due to changes in available light, the light intensity was held constant at 35 µmol m^−2^ s^−1^ (an intensity and spectral distribution equivalent to 30 min post-sunrise/pre-sunset) for the duration of all ΔF/F_m_′ measurements. Using measurements made under constant low irradiance, changes in ΔF/F_m_′ over time reflect adjustments in the proportion of light energy that is converted to photochemical energy by photosystem II. Although dark-adapted measurements are required to calculate non-photochemical quenching parameters, reductions in ΔF/F_m_′ indicate a decline in the amount of energy available for electron transport, while often implying an increase in the proportion of energy lost as heat or fluorescence^23^. Dark-adapted maximum quantum yield and ΔF/F_m_′ are in close agreement at dawn and dusk from a lack of non-photochemical quenching at low-light intensities^23^. Therefore, the measurements of ΔF/Fm′ reported here may be regarded as low-light-adapted, and interpreted as more similar to dark-adapted measurements than light-adapted measurements made under higher light intensities.

Coral condition and photosynthetic efficiency measurements were made at the beginning and end of the pre-exposure period in order to establish baseline condition of each coral used in the exposures. During the exposure, coral condition and percent recent mortality were concurrently assessed hourly for the first 8 h after exposure initiation, and every 12 h thereafter for the remainder of the 48-h exposure. Photosynthetic efficiency was also measured immediately after the exposures. Coral condition and photosynthetic efficiency of the surviving/remaining corals were subsequently assessed at 1 week post-exposure to assess the potential for recovery after exposure to 1-methylnaphthalene.

### Hydrocarbon chemistry

Water samples were collected (with no headspace) in certified volatile organic analyte vials (Thermo Scientific) from a port on the outflow line of each chamber. Samples were collected at the start (0 h, immediately prior to addition of corals), and end (48 h, immediately prior to removal of corals) of the exposure to verify the stability of the concentration throughout the exposure. After collection, samples were preserved at 4 °C, and the concentration of 1-methylnaphthalene was quantified fluorometrically with a Horiba Aqualog spectrofluorometer (λ_ex_ = 275 nm and λ_em_ = 321.5 nm). The method used for determination of 1-methylnaphthalene (1-MN) in seawater was based on SOP-2011-O-120.4, created and validated at the Environmental Analysis Research Laboratory (EARL) at Florida International University (FIU). Method details are available in the GRIIDC dataset for each experiment^24–28^.

Water samples for general water quality were collected at the start and end of the exposure. Nutrients [ammonia (NH_3_), nitrite (NO_2_), nitrate (NO_3_), and phosphate (PO_4_)] were measured with a HACH DR850 colorimeter; pH, dissolved oxygen (DO) and temperature were measured with a YSI 556 Multiprobe System; and alkalinity was determined by potentiometric titration with a Mettler-Toledo DL22 autotitrator.

### Statistical analysis

Threshold concentrations were determined with the *drc* package in R statistical software (R V3.4.3)^29^. The log-logistic 4 parameter dose response model was used for effect (EC50_Condition_) based on coral condition scores and inhibition (IC50_Yield_) based on photosynthetic efficiency. For coral condition, the individual scores for each criterion were summed and divided by the total maximum score possible, to obtain a single percent effect at each time point for each coral fragment. The percent effect for each coral fragment was averaged to determine a percent effect for each chamber. The EC50_Condition_ was then estimated from the regression of the mean percent effect and the concentration of 1-MN in each chamber, with the maximum effect fixed at 100% a priori; thus the EC50_Condition_ represents a 50% coral condition score. To estimate the concentration that inhibited photosynthetic efficiency by 50% (IC50_Yield_), the light-adapted effective quantum yield (ΔF/F_m_′) measured for each experimental time period was used to estimate the IC50_Yield_. The minimum effect level was assumed to occur in the control corals of each species (the ΔF/F_m_′ of the controls at the end of the exposure period was not different from pre-exposure levels), while the maximum potential decline in ΔF/F_m_′ was assumed to be 100%, and was fixed at 0. The log-logistic 2 parameter dose response model for binary data was used for mortality (LC50). Both models have self-starting functions that initially estimate the model parameters using the maximum likelihood principle. Estimates of all threshold levels were made with the effect dose (ED) function, which utilizes the delta method to estimate 95% confidence intervals.

The TLM was used to calculate a CTLBB_Sublethal_ and CTLBB_Lethal_ following determination of the acute and subacute endpoints for each species^11^. Ideally, the CTLBB for each species is determined by fitting the TLM relationship to endpoints from three individual hydrocarbon exposures and reducing residual error, as values estimated from one endpoint rely heavily on the universal narcosis slope and linear relationship with the chemical’s *K*_ow_. However, CTLBBs calculated from a single compound provided preliminary estimates of sensitivity for these five coral species that are useful for species comparisons and predictions of mixture toxicity.

All data were tested for normality (Komolgorov–Smirnov/Lilliefors) and homoscedasticity (Brown–Forsythe) and transformed to meet these assumptions where applicable, or nonparametric methods were used. Tukey’s unequal N honest significant difference test (parametric) or multiple comparisons (nonparametric) were used for post-hoc analysis. All statistical tests were performed using the software package STATISTICA 13. Kruskal–Wallis analysis of variance (ANOVA) on ranks (α = 0.05) with untransformed data was used to compare coral condition score (mean of 3 coral fragments in each replicate, n = 4 replicates) between treatments (during pre-exposure, exposure and post-exposure periods) and water quality data between treatments. One-way ANOVA (α = 0.05) was used to compare mean effective quantum yield (ΔF/F_m_′) between treatments (mean of 4 measurements per coral, 3 coral fragments in each replicate, n = 4 replicates) during pre-exposure, exposure and post-exposure periods. Two random coral fragments per replicate were fixed for histological and transcriptomic analysis at the end of the exposure period, therefore post-exposure recovery analyses were based on 1 coral per replicate.

## Results

### Hydrocarbon chemistry and water quality

The measured mean concentrations of 1-MN over the exposure period for each coral species and treatment are shown in Table 1. In each exposure, the concentration of 1-MN was stable over time, with average chamber coefficients of variation (CVs) of 7.4% (*A. cervicornis*), 1.9% (*P. astreoides*), 5.4% (*S. siderea*), 5.1% (*S. intersepta*), and 3.4% (*S. bournoni*) for each test. Average replicate CVs of 1.7% (*A. cervicornis*), 0.9% (*P. astreoides*), 1.0% (*S. siderea*), 0.8% (*S. intersepta*), 0.7% (*S. bournoni*), indicated high consistency in average aqueous concentrations amongst treatment replicates. Similar to previous experiments utilizing passive dosing^16,19^, the present study demonstrates the value of this methodology in achieving and maintaining stable hydrocarbon concentrations during dosing experiments^14^.

**Table 1:** Mean (± s.d.) (n = 4) concentrations of 1-methylnaphthalene, for each nominal treatment level in each coral species exposure.

Mean measured concentrations of 1-methylnaphthalene (µg L^−1^) per treatment						
Species	Control	1000 µg L^−1^	2000 µg L^−1^	4000 µg L^−1^	8000 µg L^−1^	16,000 µg L^−1^
*Acropora cervicornis*	BD	745 (± 27)	1501 (± 29)	2775 (± 134)	5370 (± 216)	9434 (± 247)
*Porites astreoides*	BD	1522 (± 39)	2868 (± 52)	5236 (± 16)	8293 (± 225)	12,530 (± 216)
*Siderastrea siderea*	BD	828 (± 13)	1614 (± 37)	3030 (± 43)	5876 (± 194)	10,332 (± 137)
*Solenastrea bournoni*	BD	788 (± 7)	1719 (± 29)	3081 (± 29)	5712 (± 138)	10,293 (± 118)
*Stephanocoenia intersepta*	BD	805 (± 14)	1616 (± 33)	2955 (± 31)	5610 (± 110)	9019 (± 102)

*BD *below laboratory detection limit of 100 µg L^−1^.

A summary of general water quality parameters is given in Supplementary Table S3. No significant differences in temperature were found between treatment chambers at the end of the exposure period (*p* > 0.05) for any of the experiments. The temperature was lower overall during the experiment with *S. siderea*, which may have affected observed impacts to this coral species. Significant differences (*p* > 0.05) in nutrient concentrations (PO_4_, NH_3_, NO_2_ and NO_3_) were not found between treatments for *P. astreoides*, *S. siderea*, *S. intersepta* or *S. bournoni*. For *A. cervicornis*, NO_2_ and NO_3_ were found to be significantly higher in the 9434 µg L^−1^ 1-MN treatment compared to the control and 745 µg L^−1^ 1-MN treatments. No significant difference (*p* > 0.05) in alkalinity between treatments was found for *S. bournoni*, however significant differences in alkalinity (*p* < 0.05) were found for *A. cervicornis*, *P. astreoides, S. intersepta*, and *S. siderea*, with alkalinity significantly higher in the high or two highest 1-MN concentrations compared to controls for *A. cervicornis*, *P. astreoides,* and *S. siderea*, potentially due to reduced calcification under high hydrocarbon concentration conditions.

Significantly lower (*p* < 0.05) pH and DO at the end of the 48 h exposures were found in the highest 1-MN treatments compared to the control and/or low concentration treatments for all species. However, all DO measurements, except for the highest test concentrations for *A. cervicornis* and *P. astreoides*, were within 10% of the saturation or control concentration at 48 h. Similarly, pH was within 0.18 pH units of the control at 48 h except for the highest test concentration for *A. cervicornis*. As this was observed in a previous experiment^16^, pH and DO measurements were also taken at 24 h. As pH at 24 h was within 0.13 pH units of the control, and DO at 24 h was within 30% of the saturation or control concentration, the observed decreases in pH and DO at 48 h were concluded to be the result of coral tissue necrosis or decomposition of released mucus which occurred between 12 and 24 h of exposure in *A. cervicornis* and *P. astreoides* in the 9434 µg L^−1^ 1-MN and 12,530 µg L^−1^ 1-MN treatments, respectively. The limited visual changes in health observed in the control corals (for any of the tested species) indicated the minimal impact of the closed exposure system design on coral health for short time periods.

### Coral condition

**Figure 1:**
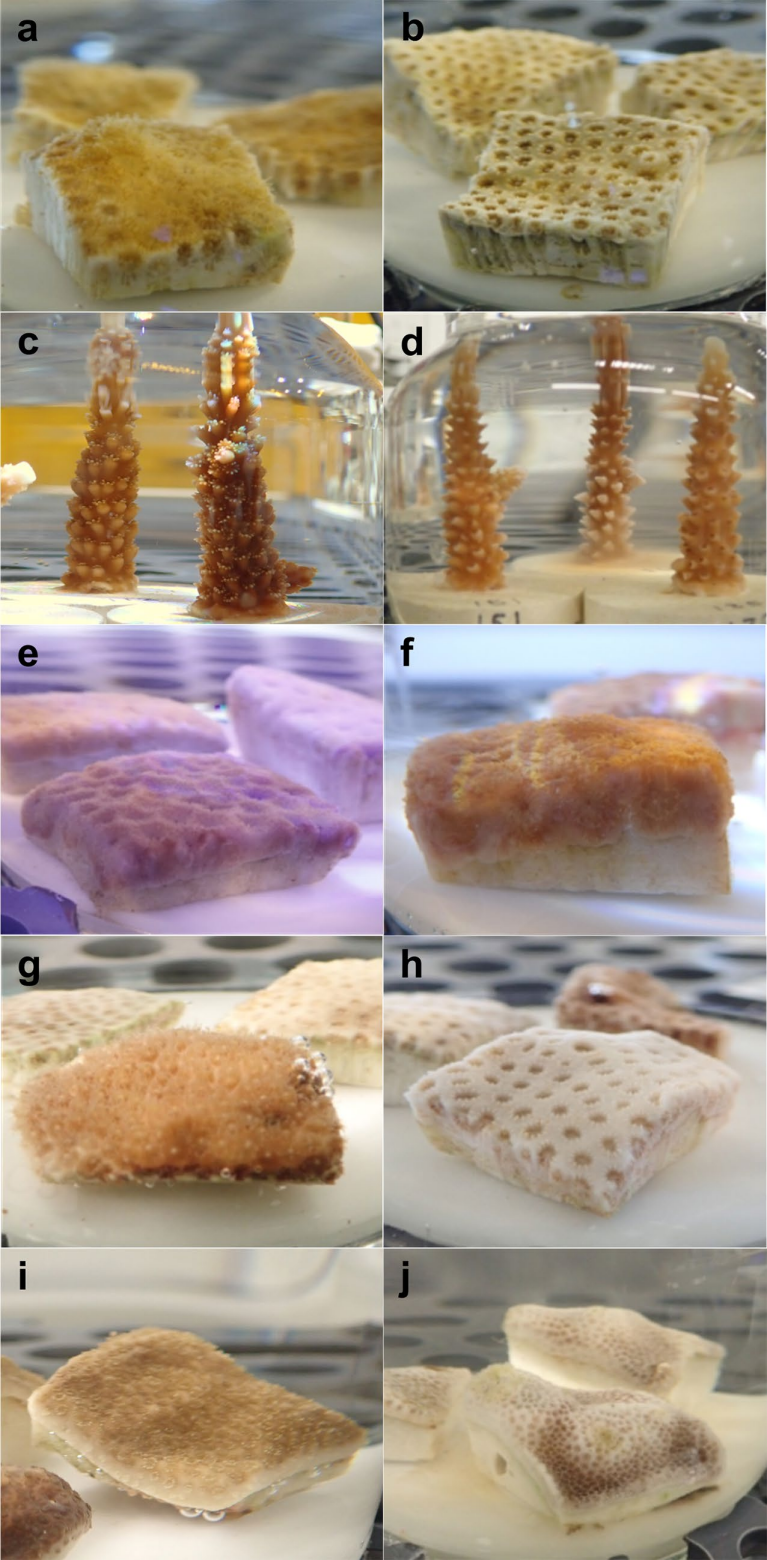
Typical physical changes at various time points in response to 1-methylnaphthalene (1-MN) exposure. (**a**) *Solenastrea bournoni*, control coral @ T1, and (**b**) polyp retraction in the 10,293 µg L^−1^ 1-MN treatment @ T1; (**c**) *Acropora cervicornis,* control coral @ T12, and (**d**) elevated mucus production in the 9434 µg L^−1^ 1-MN treatment @ T12; (**e**) *Siderastrea siderea,* control coral @ T8, and (**f**) tissue swelling in the 3030 µg L^−1^ 1-MN treatment @ T8; (**g**) *Stephanocoenia intersepta,* control coral @ T36, and (**h**) bleaching in the 1616 µg L^−1^ 1-MN treatment @ T36; and (**i**) *Porites astreoides*, control coral @ T36, and (**j**) tissue loss/mortality in the 12,530 µg L^−1^ 1-MN treatment @ T36.

The corals exhibited a range of responses to 1-MN exposures, with a degree of variability between species. Control corals maintained normal polyp extension and mucus production, with no tissue swelling. Corals exposed to low 1-MN concentrations (or high concentrations for over short time scales) exhibited polyp retraction compared to controls (Fig. 1a,b, in *S. bournoni*) and elevated mucus production (Fig. 1c,d, in *A. cervicornis*). Tissue swelling, typically of the coenenchyme, was also frequently observed in response to moderate hydrocarbon concentrations (Fig. 1e,f, in *S. siderea*). At higher concentrations, response included tightly retracted polyps, followed by lightening of coloration and bleaching (Fig. 1g,h, in *S. intersepta*). Highly stressed corals had severe polyp retraction, with degradation of the coenenchyme, exposure of skeletal elements and tissue loss or mortality (Fig. 1i,j, in *P. astreoides*).

In *A. cervicornis*, severe polyp retraction occurred in the 5370 µg L^−1^ 1-MN and 9434 µg L^−1^ 1-MN corals within 1 h of exposure, which progressed to moderate to severe tissue attenuation after 12 h of exposure. Severe lightening of color (bleaching) was not observed in the high 1-MN concentrations before the onset of mortality at 12 h, but mild to moderate bleaching was observed in many of the mid-concentration corals after 12 h. Elevated mucus production was observed in the high 1-MN concentrations after 3 h, and progressed to very high levels after 12 h. Limited tissue swelling was observed in this species.

A similar, but less severe pattern was observed in *P. astreoides*. Mild to moderate polyp retraction was observed in concentrations > 1522 µg L^−1^ after 1 h of exposure. Mucus production in the 12,530 µg L^−1^ corals was mild to moderate after 4 h, progressing to severe after 5 h. Mild to moderate bleaching was observed in the 12,530 µg L^−1^ corals after 7 h. Mild to moderate swelling of the coenenchyme was observed in corals exposed to ≥ 2868 µg L^−1^ after 7 h, which progressed to mild to moderate tissue thinning in the two highest 1-MN concentrations after 8 h. After 1 week of recovery, most corals in all treatments improved to pre-exposure levels, but moderate to severe polyp retraction and bleaching were still evident in some corals exposed to high 1-MN concentrations.

Less severe, sublethal effects were observed in the other three species. In *S. intersepta*, mild to moderate polyp retraction was observed in the 5610 µg L^−1^ 1-MN and 9019 µg L^−1^ 1-MN corals after 1 h, and in all concentrations ≥ 1616 µg L^−1^ 1-MN after 3 h, along with mild tissue swelling and mucus production. Moderate to severe tissue attenuation and bleaching was progressively observed in the two highest 1-MN concentrations from 24 to 48 h. In *S. siderea*, mild tissue swelling and mucus production was observed in concentrations > 3030 µg L^−1^ 1-MN after 2 h. Mild to moderate polyp retraction was observed in concentrations > 828 µg L^−1^ 1-MN after 1 h of exposure, which progressed to severe polyp retraction and moderate tissue attenuation by 8 h in the 10,332 µgL 1-MN corals, along with mild to moderate bleaching. Severe attenuation and bleaching were observed after 48 h in the 10,332 1-MN µg L^−1^ corals. Moderate to severe bleaching, polyp retraction and tissue attenuation was observed in the two highest 1-MN concentrations after 48 h. In the least affected coral, S. *bournoni*, mild to moderate polyp retraction was observed in concentrations > 1719 µg L^−1^ 1-MN after 1 h of exposure. Mild to moderate tissue swelling was observed in the 5712 µg L^−1^ 1-MN and 10,293 µg L^−1^ 1-MN corals after 5 h, progressing to mild tissue attenuation after 24 h in the highest concentration.

**Figure 2:**
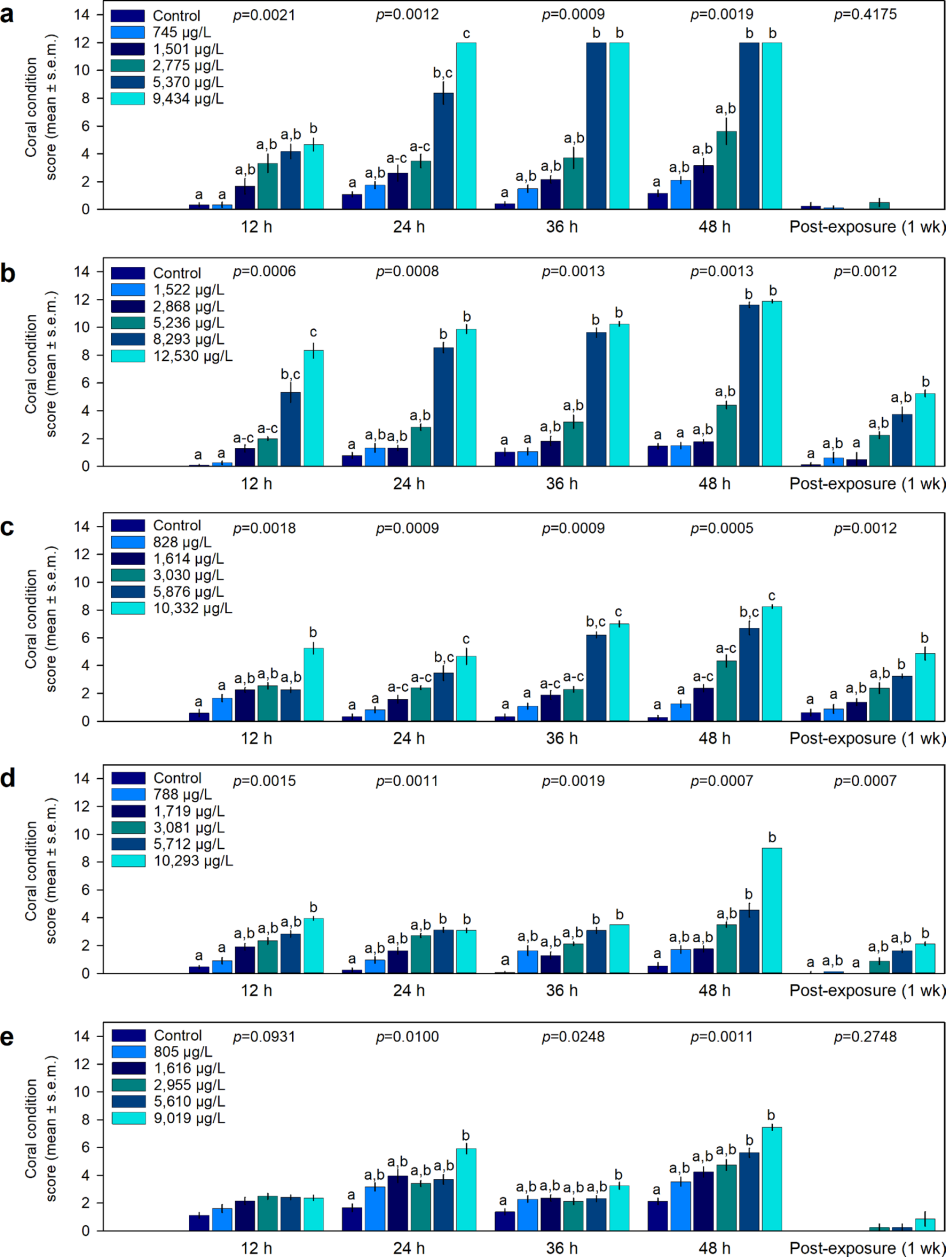
Coral condition scores (mean ± s.e.m.) (n = 4 replicates) during 1-methylnaphthalene exposures at 12 h, 24 h, 36 h, 48 h and 1 week post-exposure. (**a**) *Acropora cervicornis*, (**b**) *Porites astreoides*, (**c**) *Siderastrea siderea,* (**d**) *Solenastrea bournoni*, and (**e**) *Stephanocoenia intersepta*. Letters above each bar represent statistical differences between treatments at each time point (**a**–**c**; α = 0.05).

Comparison of coral condition scores for each treatment, at each interval during the exposure found significant treatment effects (Kruskal–Wallis ANOVA, *p* < 0.05, Supplementary Table S4) from 1 h after initiation until the end of the exposure for all coral species (Fig. 2). For *A. cervicornis*, post-hoc analysis indicated that the 5370 µg L^−1^ 1-MN and higher treatment corals scored significantly higher (*p* < 0.05) than the 2775 µg L^−1^ 1-MN and lower treatments after 2 h of exposure (Supplementary Table S4), with comparable response seen throughout the remainder of the exposure period (Fig. 2a). A similar pattern of response was seen for *P. astreoides* (Fig. 2b), *S. siderea* (Fig. 2c), *S. bournoni* (Fig. 2d), and *S. intersepta* (Fig. 2e).

Recovery was evident in all species after 1 week post-exposure, although significant differences (p < 0.05) in coral condition, resulting from lasting mild to moderate polyp retraction and bleaching, were found in *P. astreoides* (Fig. 2b), *S. siderea* (Fig. 2c), and *S. bournoni* (Fig. 2d). No significant differences (p > 0.05) were found between treatments for any species after 4 weeks (Supplementary Table S4).

### Photosynthetic efficiency

**Figure 3:**
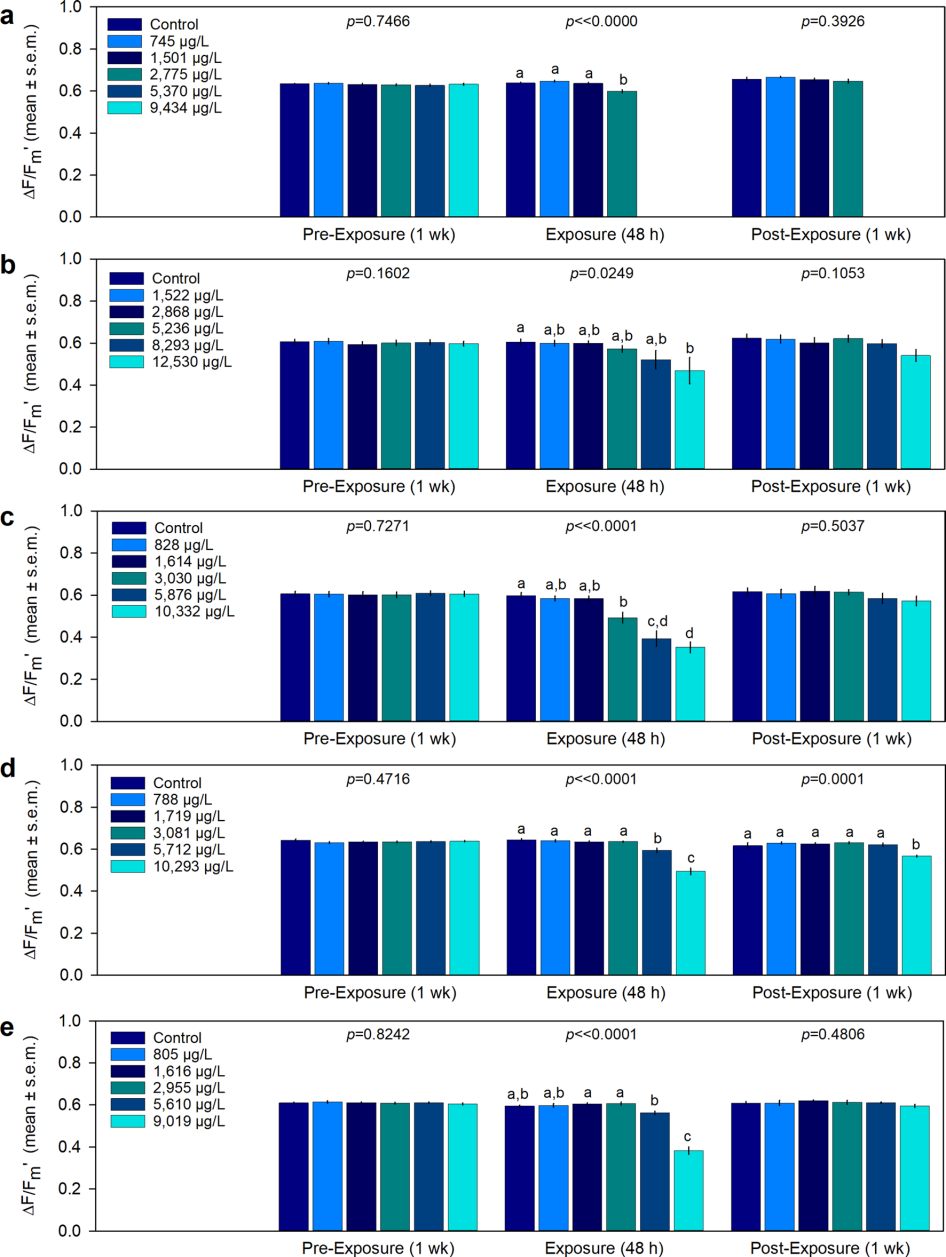
Light-adapted effective quantum yield (mean ± s.e.m.) (n = 4 replicates) during the pre-exposure, exposure, and post exposure periods. (**a**) *Acropora cervicornis*, (**b**) *Porites astreoides*, (**c**) *Siderastrea siderea,* (**d**) *Solenastrea bournoni*, and (**e**) *Stephanocoenia intersepta*. Letters above each bar represent statistical differences between treatments at each time point (**a**–**c**; α = 0.05).

Mean ΔF/F_m_′ (Fig. 3) was not significantly different (One-way ANOVA, *p* > 0.05, Supplementary Table S5) between treatments at the end of the pre-exposure periods for all species. For *P. astreoides* and *S. intersepta*, ΔF/F_m_′ in the highest concentration tested (12,530 µg L^−1^ 1-MN and 9019 µg L^−1^ 1-MN, respectively) was significantly less than the control corals after the exposure period (Fig. 3b,e). In *S. bournoni*, ΔF/F_m_′ in the two highest tested concentrations, 5712 µg L^−1^ 1-MN and 10,293 µg L^−1^ 1-MN, was significantly less (*p* < 0.05) than all lower concentrations, with no difference found between the controls or corals exposed to concentrations ≤ 3081 µg L^−1^ 1-MN (*p* > 0.05) (Fig. 3d). For *S. siderea*, significant reductions (*p* < 0.05) in ΔF/F_m_′ in the three highest concentrations tested (3030–10,332 µg L^−1^ 1-MN) compared to the controls were observed (Fig. 3c). Mortality in the two highest concentrations tested for *A. cervicornis* precluded these comparisons, however mean ΔF/F_m_′ in the corals exposed to 2775 µg L^−1^ 1-MN was significantly less (*p* < 0.05) than all other treatments at the end of the exposure period (Fig. 3a).

After 1 week of recovery, significant differences in ΔF/F_m_′ between treatments were only found in *S. bournoni*, where the corals exposed to the highest concentration tested (10,293 µg L^−1^ 1-MN) were significantly less (*p* < 0.05) than all other treatment corals (Fig. 3d). No significant differences (*p* > 0.05) were found between treatments for any species after 4 weeks (Supplementary Table S5).

### Coral mortality

**Figure 4:**
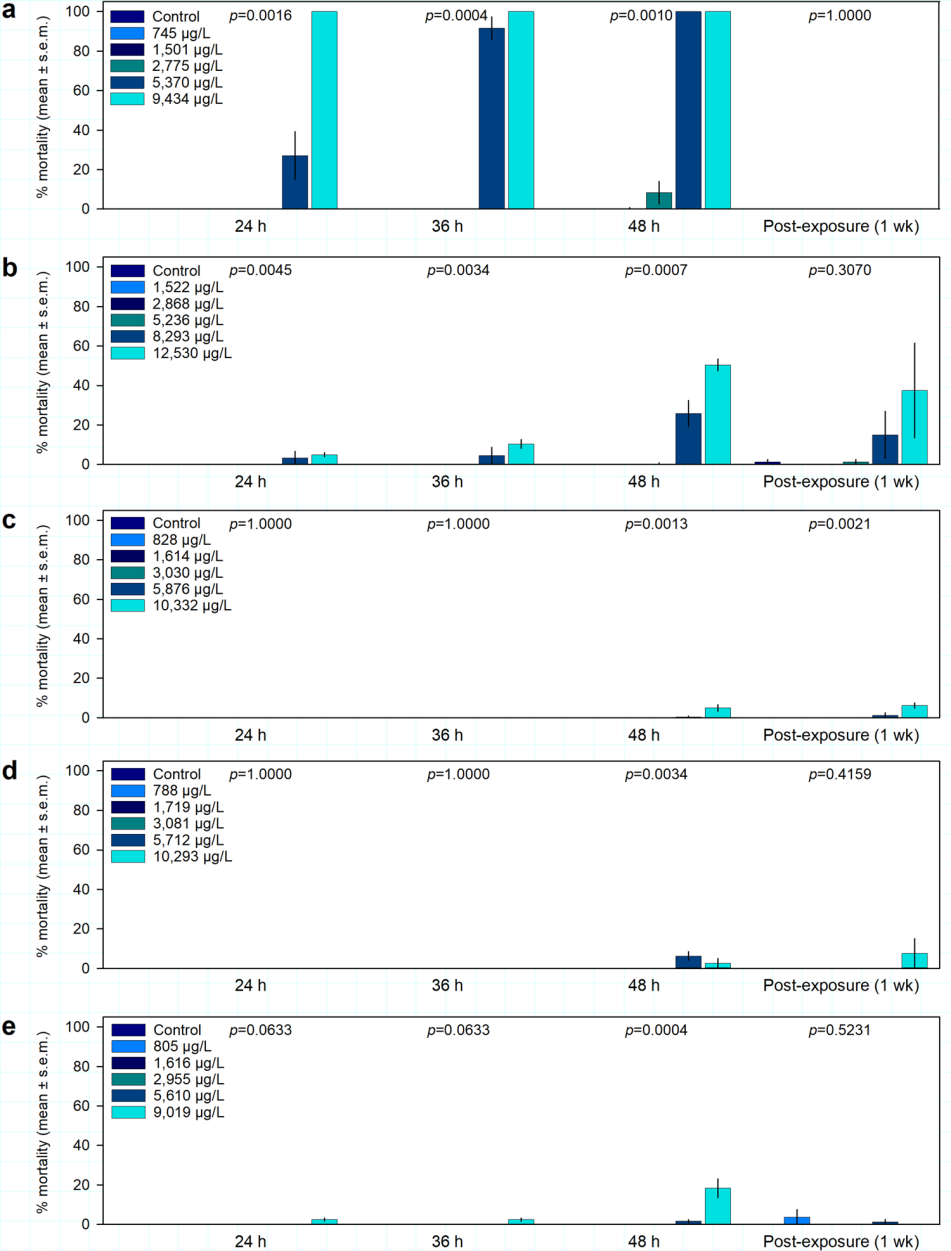
Coral percent mortality (mean ± s.e.m.) (n = 4 replicates) during 1-methylnaphthalene exposures at 24 h, 36 h, 48 h of exposure and at 1 week post-exposure. (**a**) *Acropora cervicornis*, (**b**) *Porites astreoides*, (**c**) *Siderastrea siderea,* (**d**) *Solenastrea bournoni*, and (**e**) *Stephanocoenia intersepta*.

The percent mortality observed over time as a result of exposure to 1-MN was variable between species, with significant treatment impacts (Kruskal–Wallis ANOVA, *p* < 0.05, Supplementary Table S6) at 24 h, 36 h, and 48 h in *A. cervicornis* and *P. astreoides* (Fig. 4). In *A. cervicornis*, 100% mortality was observed in the 9434 µg L^−1^ 1-MN treatment within 24 h of exposure; in the 5370 µg L^−1^ treatment, 27% (± 12%) was observed after 24 h, 92% (± 5.6%) after 36 h, and 100% after 48 h; and 8.3% (± 5.6%) mortality in the 2775 µg L^−1^ treatment after 48 h (Fig. 4a). Mortality was also observed in *P. astreoides*, with 50.4% (± 7.9%) mortality in the 12,530 µg L^−1^ 1-MN treatment and 25.8% (± 9.6%) mortality in the 8293 µg L^−1^ 1-MN treatment after 48 h of exposure (Fig. 4b). Comparatively lower mortality occurred in *S. siderea* (5.0% ± 1.6%) and *S. intersepta* (18.3% ± 4.37%) at the highest concentrations tested, with significant treatment impacts (*p* < 0.05) only observed at 48 h (Fig. 4c,e). Very limited mortality was observed in *S. bournoni* after 48 h of exposure, with 6.2% (± 2.1%) in the 5712 µg L^−1^ 1-MN treatment and 2.5% (± 2.5%) in the 10,293 µg L^−1^ 1-MN treatment (Fig. 4d).

Recovery was observed in all species, with significant treatment effects after 1 week post-exposure only in *S. siderea* (Fig. 4c). No significant differences (*p* > 0.05) were found between treatments for any species after 4 weeks (Supplementary Table S6).

### Acute and subacute thresholds

**Figure 5:**
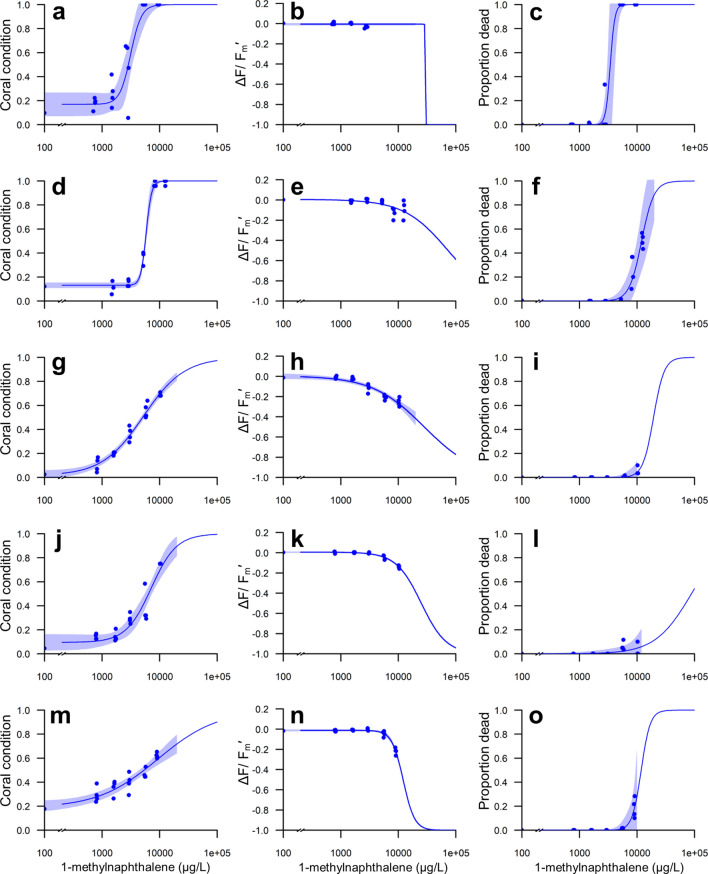
Dose–response curves for the effects of 48 h 1-methylnaphthalene exposure on coral condition (left column), light-adapted effective quantum yield (ΔF/F_m_′) (middle column), and mortality (right column). (**a**–**c**) *Acropora cervicornis*, (**d**–**f**) *Porites astreoides*, (**g**–**i**) *Siderastrea siderea,* (**j**–**l**) *Solenastrea bournoni*, and (**m**–**o**) *Stephanocoenia intersepta*.

Coral response was used to estimate acute and subacute endpoints for sublethal effects and mortality; specifically, EC50_Condition_ was calculated from the coral condition score, IC50_Yield_ was calculated from ΔF/F_m_′, and LC50 was calculated from percent mortality. Dose–response curves for each species following 48 h exposure to 1-MN are shown in Fig. 5. The subacute effects on coral condition for *A. cervicornis* indicated a significant positive relationship between the concentration of 1-MN and the increasing effect observed (*p* < 0.001), producing the lowest EC50_Condition_ (3126 µg L^−1^ 1-MN) for all coral species examined here (Fig. 5a). Exposure to 1-MN caused a decline in ΔF/F_m_′ compared to controls in *A. cervicornis* (max decline of 9.8%, 0.07 units), and resulted in an IC50_Yield_ of 7384 µg L^−1^ 1-MN, which is above the highest surviving concentration of 1-MN (Fig. 5b) and thus largely related to mortality. Acute effects were used to determine an LC50 of 3421 µg L^−1^ 1-MN (Fig. 5c).

The subacute impacts of 1-MN exposure in *P. astreoides* were comparatively less severe than in *A. cervicornis*, but resulted in an EC50_Condition_ of 5819 µg L^−1^ 1-MN (Fig. 5d). Declines in ΔF/F_m_′ following exposure to 1-MN were observed in the highest concentrations, but did not result in an IC50_Yield_ within the solubility of 1-MN in seawater (Fig. 5e) as the maximum observed decline was 35% (0.22 units). Acute impacts were used to estimate an LC50 of 11,982 µg L^−1^ 1-MN for *P. astreoides* (Fig. 5f).

Subacute effects on coral condition in *S. siderea* resulted in an EC50_Condition_ of 5189 µg L^−1^ 1-MN (Fig. 5g), and declines in ΔF/F_m_′ (max decline of 50%, 0.32 units), resulted in an IC50_Yield_ of 12,378 µg L^−1^ 1-MN (Fig. 5h), which was above the highest 1-MN concentration tested. These subacute impacts indicated that *S. siderea* was less sensitive than *A. cervicornis*, but more sensitive than *P. astreoides*. Mortality in *S. siderea* following 1-MN exposure was below the level required to calculate an LC50 at the maximum measured concentrations (Fig. 5i).

Similarly, mortality in *S. bournoni* following 1-MN exposure (Fig. 5l) was not sufficient to calculate an LC50 at the maximum measured 1-MN concentration. Subacute effects of 1-MN on *S. bournoni* were used to estimate an EC50_Condition_ of 7127 µg L^−1^ 1-MN (Fig. 5j). The decline in ΔF/F_m_′ (29%, 0.19 units) resulted in an IC50_Yield_ of 17,799 µg L^−1^ 1-MN (Fig. 5k), which was above the highest 1-MN concentration tested.

Subacute effects of 1-MN exposure in *S. intersepta* occurred at higher concentrations when compared to the previously described species, with an EC50_Condition_ of 9294 µg L^−1^ 1-MN (Fig. 5m). The decline in ΔF/F_m_′ (43%, 0.27 units) resulted in an IC50_Yield_ of 10,173 µg L^−1^ 1-MN (Fig. 5n), which is between the estimated EC50 and the LC50 of 11,787 µg L^−1^ (Fig. 5o).

The 48-h EC50s and LC50s determined from the dose–response curves for each species were input into the TLM to calculate a corresponding CTLBB_Sublethal_ and CTLBB_Lethal_ for each coral species (Table 2). Toxicity is time dependent, and longer exposures lead to a reduction in calculated threshold concentrations due to the accumulation of toxicant over time, until tissues reach the incipient effect level for that organism^30^. The incipient E/LC50 (E/LC50_∞_) is *K*_ow_ dependent, and volatile and semi-soluble PAHs with a relatively low *K*_ow_ such as 1-MN have been shown to reach incipient levels by 48 h in other organisms^31^. Calculated EC50_Condition_ and LC50 endpoints (provided in Supplementary Table S7) show a slight decline after 24 h for each species, suggesting that the threshold concentrations calculated at 48 h were approaching incipient levels, and may be representative of incipient thresholds for these coral species. The CTLBBs calculated from the E/LC50s measured here are likely similar to, but slightly greater than the CTLBBs that would be calculated from incipient thresholds.

**Table 2:** Estimates of IC50_Yield_, EC50_Condition_, and LC50 (± respective 95% CIs) for 1-methylnaphthalene and associated calculated critical target lipid body burdens (± 95% CI) (µmol g^−1^).

Species	Time (h)	IC50_Yield_ (µg L^−1^)	EC50_Condition_ (µg L^−1^)	CTLBB_Sublethal_	LC50 (µg L^−1^)	CTLBB_Lethal_
*Acropora cervicornis*	48	7384 (2525–12,243)	3126 (2573–3678)	182 (150–214)	3421 (2670–4174)	199 (156–243)
*Porites astreoides*	48	> Solubility	5819 (5594–6045)	339 (326–532)	11,982 (CNC)	698 (CNC)
*Siderastrea siderea*	48	12,378 (9396–15,360)	5189 (4583–5794)	302 (26–337)	> Solubility	–
*Solenastrea bournoni*	48	17,799 (14,611–20,987)	7127 (5945–8310)	415 (346–484)	> Solubility	–
*Stephanocoenia intersepta*	48	10,173 (9737–10,608)	9294 (6370–12,217)	541 (371–712)	11,787 (4956–18,618)	687 (286–1084)

*CNC *could not calculate.

### Comparative toxicity

**Figure 6:**
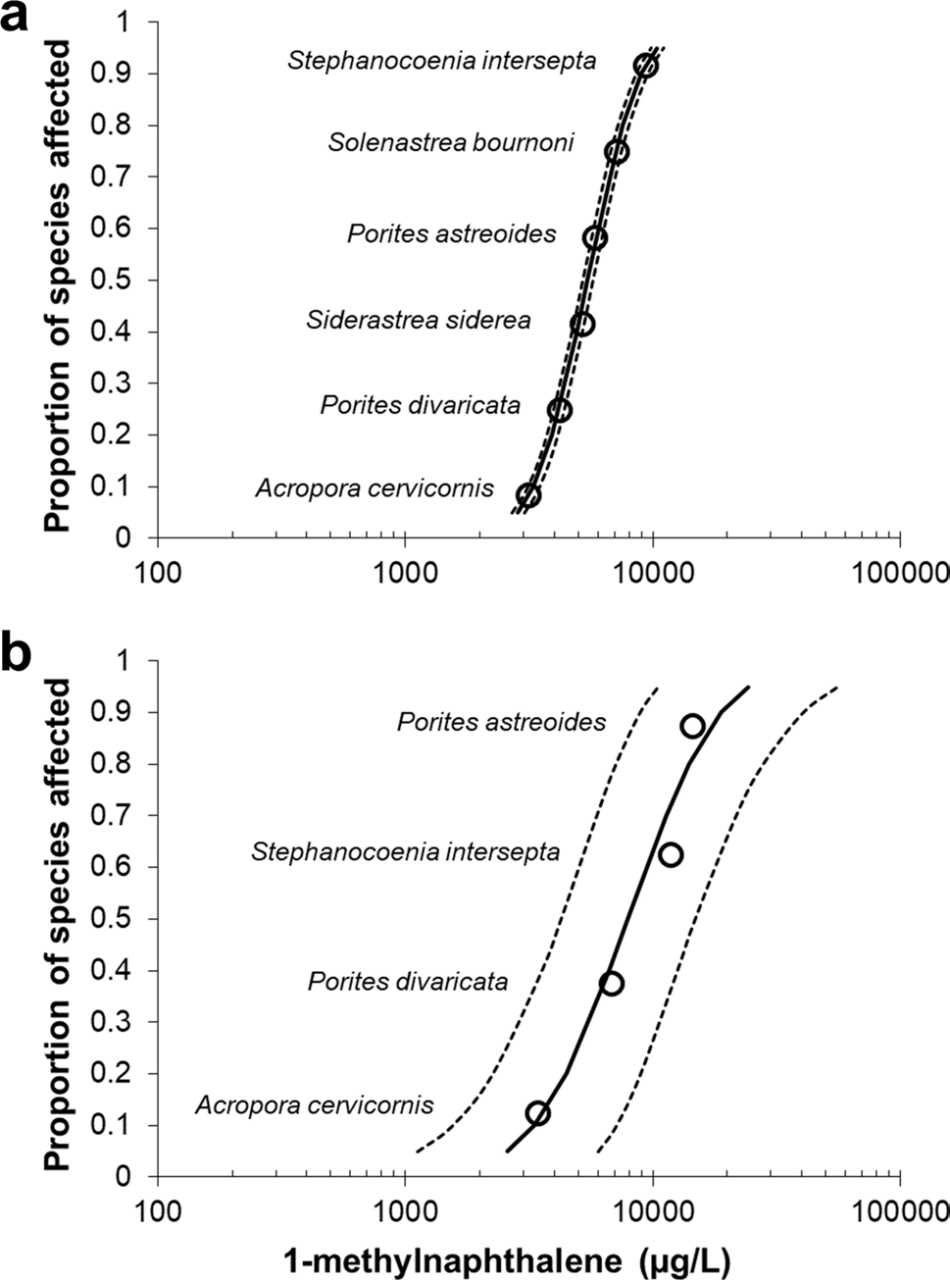
Species sensitivity distributions for (**a**) 1-methylnaphthalene 48-h EC50s and (**b**) 1-methylnaphthalene 48-h LC50s. Endpoints for *Porites divaricata* from Renegar et al.^36^.

To assess relative sensitivity, the subacute and acute endpoints for these corals were used to generate species sensitivity distributions for 1-MN (48-h EC50s, Fig. 6a, and 48-h LC50s, Fig. 6b). The ESA threatened species *A. cervicornis* was overall the most sensitive to petroleum hydrocarbon exposure for each endpoint. However, these SSDs must be interpreted with caution, as a larger number of species are needed to minimize variability and imprecision in toxicological benchmarks such as the HC5^32,33^.

**Figure 7:**
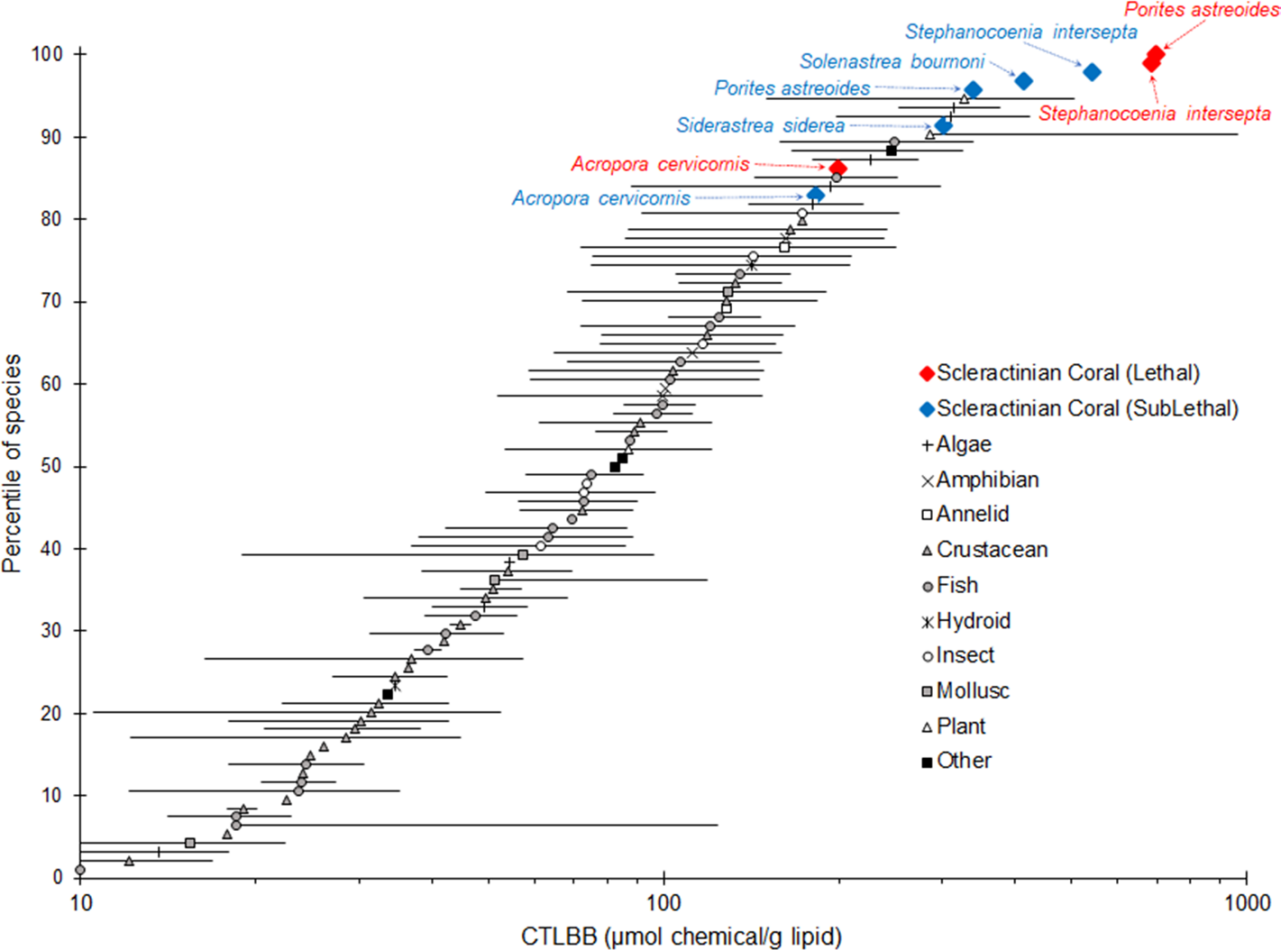
Comparison of critical target lipid body burdens (CTLBBs) (µmol chemical/g lipid) of 94 taxa, including freshwater, brackish, and marine species^18,34,35^.

The CTLBB_Sublethal_ and CTLBB_Lethal_ (Table 2) calculated for *A. cervicornis*, *P. astreoides*, *S. siderea*, and *S. intersepta,* and *S. bournoni* and were compared to calculated values for 94 freshwater, brackish water, and marine species (Fig. 7). Although these CTLBBs are calculated from one hydrocarbon and should therefore be interpreted as preliminary, the CTLBB_Sublethal_ ( ) and CTLBB_Lethal_ ( ) for these scleractinian coral species indicate that they are comparatively more resilient to narcotic chemical exposure compared to other species for which similar data is available^18,34,35^. The CTLBB_Sublethal_ for the most sensitive species tested here, *A. cervicornis*, falls at the 82nd percentile. If compared to 48 marine species, the CTLBB_Sublethal_ for *A. cervicornis* falls at the 83rd percentile (not shown).

## Discussion

The objective of this research was to develop a more complete understanding of petroleum hydrocarbon toxicity to shallow-water scleractinian corals, by utilizing a standardized test protocol to determine scientifically defensible toxicity benchmarks for multiple Atlantic coral species. The tested corals were found to have a variable range of species-specific physical responses to petroleum hydrocarbon exposures, with *A. cervicornis* the most sensitive overall. Significant dose-dependent physical impacts were observed in each coral species exposed to 1-MN, progressing from initial observations of polyp retraction and tissue distension to eventual tissue thinning (with exposure of skeletal elements) and necrosis. These observed impacts were consistent with previous studies of petroleum hydrocarbon impacts on adult corals^16,37–40^. A degree of recovery from sublethal physical and photophysiological changes was observed after 1 week post-exposure for surviving corals of all species, with progressive improvement throughout the 4 week post-exposure period.

The expansion and retraction of polyps is a key behavior in cnidarians^41^. Polyp expansion is important to several processes, including feeding, gas exchange, and diffusion of waste products^42–45^. Polyp retraction is employed in predation avoidance (the startle response) as well as several metabolic processes^41,42,46^, and has been frequently observed as a response in corals exposed to hydrocarbons^5,47,48^. In this study, polyp retraction was the primary response to increasing levels of 1-MN exposure in all of the tested species, and occurred quickly, with severe retraction occurring in *A. cervicornis* and *P. astreoides* within 1 h and 5 h (respectively) of exposure to high concentrations of 1-MN. This level of polyp retraction represented a significant level of coral stress that was not immediately reversible, and progressed to tissue attenuation and mortality in these two species. Mucus production was also an important initial response observed to 1-MN exposure; increased mucus production is frequently associated with environmental stress and exposure to pollutants, and may allow corals to efficiently and actively depurate lipophilic chemicals such as hydrocarbons^37,49^. As such, elevated mucus production is a common observation during stressor experiments with corals, including when exposed to hydrocarbons^16,37,50,51^. Elevated mucus production during exposure to 1-MN was observed at varying degrees of severity in all species tested here, however the largest amount of mucus release caused by 1-MN exposure occurred in *A. cervicornis* and *P. astreoides*.

Swelling of polyps and tissues, or hypertrophy, is also common observation related to different types of stress in corals^52–54^, and is likely related to an increase in the size and number of mucocytes and mesogleal swelling^21,37^. Varying degrees of tissue swelling are noted in corals exposed to hydrocarbons^16,37,52^, and severe swelling can be followed by tissue lysis and necrosis; tissue swelling was generally followed by tissue attenuation in these 1-MN exposures. The most severe tissue swelling occurred in *S. siderea*, with only mild to moderate swelling observed in the other species. Extreme swelling and/or polyp distension, which was previously observed in *P. divaricata* exposed to 1-MN^16^, was not observed to the same degree in the species tested here.

The measured decrease in photosynthetic yield after exposure to 1-MN has also been observed in other scleractinian coral species, although not consistently^55^. Unlike other studies which assess changes to specific parameters of coral/zooxanthellae photophysiology using dark-adapted maximum quantum yield, this study used light-adapted effective quantum yield to measure the proportion of light energy reaching PSII that is transferred to photochemical energy under constant irradiance^23^. For all 5 tested species, the observed reductions in ΔF/F_m_′ resulted in an IC50_Yield_ that was greater than the calculated EC50_Condition_. Additionally, the IC50_Yield_ was higher than the LC50 for *A. cervicornis* and greater than the solubility of 1-MN in seawater for *P. astreoides*. The impacts to ΔF/F_m_′ were therefore less severe than the visual sublethal changes in coral condition, and were not the most sensitive indicator of 1-MN toxicity in the tested coral species. A similar result was found for the Pacific coral *Pocillopora damicornis*^48^.

Acute and subacute threshold concentrations are valuable for comparing effects of the same compound, but a lack of available data may limit the applicability of species sensitivity distributions to estimate hazard concentrations^33^. The TLM was therefore used to facilitate comparison with additional species exposed to other hydrocarbons by estimating the CTLBB, which is a direct measure of chemical toxicity attributed to differences in partitioning of the chemical into the target lipid^10,56^. Typically, the TLM is fit to endpoint data from three or more hydrocarbons in order to assess the relationship between observed effects and chemical *K*_ow_. However, endpoint data for one compound can also be used to generate a preliminary CTLBB for that organism, which can then be used to assess relative sensitivity across many compounds and species. The preliminary CTLBBs determined here indicate that adults of the tested coral species are more resilient to dissolved hydrocarbon exposure compared to other taxa for which similar data is available, including other coastal marine species. Early life stages (i.e. gametes, larvae, and juveniles) of corals have been shown to be more sensitive than the adult corals, although species and contaminants were different than those used here^2,57,58^.

For single hydrocarbons such as 1-MN, the mode of action underlying baseline toxicity is narcosis, or the non-specific partitioning of chemicals in biological membranes and membrane-protein interfaces; the function of the lipid membranes is altered due to an increase in fluidity of the membranes, which accompanies solubilization of the narcotic chemical^59^. The lipid content of the organism has been observed to have a significant positive linear relationship to the acute toxicity endpoint^60,61^. This is particularly relevant to coral tissue, which has a relatively high total lipid content (≈ 8–34%), consisting of components that can vary based on multiple environmental factors^62–68^. Cnidarians have a large and diverse group of total lipids that are composed of non-polar storage lipids (wax esters and triglycerides), polar structural lipids (phospholipids), and additional symbiont (zooxanthellae) lipids. For scleractinian corals, the significant lipid storage reserves (22–32% of total) are accompanied by a large amount of structural lipid (10–18% of total)^66^ which may serve in a protective role during exposure to non-polar chemicals. Both the total and relative amounts of each lipid class can vary based on the corals’ health status and environmental conditions. Specifically, the balance between storage and structural lipids is important for species specific thermal resistance in corals, and loss of symbiotic zooxanthellae (bleaching) resulting from ocean warming can significantly reduce total coral lipid content^69,70^. Zooxanthellae densities are known to decrease due to other environmental factors as well (i.e. ocean acidification and land-based sources of pollution), which would reduce overall storage lipid ratios and cause a significant decline in coral storage lipids following increased utilization of energy reserves. Short-term exposure to elevated temperatures has also been shown to reduce polar structural lipids in the coral animal^69^. Thus, changes in the environment that lead to reductions in structural lipids could potentially lead to disruptions in normal processes at lower levels of hydrocarbon exposure. In addition to structural lipids, all corals secrete mucus, a polysaccharide protein-lipid complex which comprises the corals’ surface mucus layer (SML). The SML plays a key role in processes such as heterotrophic feeding and sediment removal, and provides a protective physiochemical barrier^71,72^. Bioaccumulation and preferential partitioning of PAHs in the SML has been found in multiple coral species, and may temporarily prevent accumulation of lipophilic chemicals in structural lipids^73^. Corals’ significant tissue lipid reserves, coupled with depuration via mucus secretion may therefore in part explain the relative resilience of corals to hydrocarbon exposure, although extended periods of acute exposure could eventually exhaust energetic reserves and result in sublethal effects or mortality.

This study has demonstrated that five species of Atlantic scleractinian corals were resilient to dissolved petroleum hydrocarbon exposure in a laboratory setting. While this method of sensitivity assessment is recommended for the evaluation of comparative risk and fate and effects modeling, it cannot fully consider the potential for environmental complexity during spill events. For example, forecast scenarios of oil spill impacts to coral reefs frequently consider the potential impacts of dispersed or non-dispersed floating oil in the sea^74^, which is subject to extensive and rapid direct and indirect photooxidation^75^. Shallow-water ecosystems are therefore particularly vulnerable to impacts from phototoxicity. The Phototoxic Target Lipid Model (PTLM)^76^ has been developed to predict the phototoxicity of single PAHs, however it does not account for the complex changes associated with rapid photooxidation of crude oil. A recent review identified the three most important potential co-stressors during oil spills as ultraviolet radiation (UVR), temperature, and pH; the limited available data indicated a 7.2 fold increase in toxicity from UVR exposure, a 3.0 fold increase in toxicity from elevated temperature, and a 1.3 fold increase in toxicity from decreased pH^58^. The potential for substantial co-impacts of these and other environmental factors, combined with the lack of definitive experimental data on the interactive effects of these factors with spilled oil, strongly supports the need for additional research to evaluate the multifaceted risk of oil spills to increasingly vulnerable coral reef ecosystems.

## Conclusions

The application of a standardized test protocol has generated new PAH toxicity endpoints for five species of Atlantic scleractinian corals, and provided the first species sensitivity distributions for scleractinian corals. The measured acute and sub-lethal endpoints indicate that the Atlantic staghorn coral, *A. cervicornis,* was the most sensitive to PAH exposure; its status as a threatened species under the Endangered Species Act is an important consideration in the oil spill response planning process. Overall, the results indicate that the tested corals are more resilient to petroleum hydrocarbon exposure than other marine species, including many fish and crustaceans. While the reason for this relative resilience is not yet fully understood, it may be related to the lipid content of coral tissue, and corals’ ability to produce lipid-rich coral mucus. As these attributes can vary substantially (i.e., temporal, inter- and intraspecific variation), additional research is needed to understand the effects of compounding environmental factors and stress, and further elucidate oil impacts and impact thresholds of petroleum hydrocarbons on scleractinian corals.

Species-specific CTLBB estimates were generated in order to provide the preliminary data needed to predict oil toxicity in the environment^13,15^. This new data, when integrated into response support tools, will significantly improve the prediction of oil impacts on the coral animal and related habitats at variable severity levels. This research was designed with the goal of building a foundation for effective decision-making should a spill potentially impact coral reefs, and provides key information to be used in Net Environmental Benefit Analysis (NEBA) or Spill Impact Mitigation Assessment (SIMA) of calculated impacts of spill response methods in coral reef environments.

## Supplementary Information

Supplementary Information.

## Data Availability

Data are available through the Gulf of Mexico Research Initiative Information & Data Cooperative (GRIIDC) portal at https://data.gulfresearchinitiative.org [DOIs: 10.7266/N7NP22ZB, 10.7266/N7DF6PSG, 10.7266/n7-d2ww-0y33, 10.7266/n7-4bhj-qj29, 10.7266/n7-g2v6-0s84] and [UDIs: R6.×825.000:0001-15].
