# Supplementary material for: Species sensitivity assessment of five Atlantic scleractinian coral species to 1-methylnaphthalene
Source: Sci Rep. 2021 Jan 12;11:529. doi: 10.1038/s41598-020-80055-0 (PMC7804185; doi:10.1038/s41598-020-80055-0)
Supplement: Supplementary file 1 — Supplementary Information. [file 41598_2020_80055_MOESM1_ESM.pdf]

Species sensitivity assessment of five Atlantic scleractinian coral species to 1-methylnaphthalene

*D. Abigail Renegar[1], Nicholas R. Turner[1]

[1]Nova Southeastern University, Halmos College of Natural Science and Oceanography
Dania, FL, USA

*drenegar@nova.edu

Semi-quantitative scoring matrix for the assessment of sublethal stress in corals

To estimate sub-acute effects, specific physical characteristics of each coral are semi-quantitatively scored. The scoring system assesses relative deviations from the normal state of each species; initial range-finding experiments are very useful to establish the full spectrum of possible or expected effects in each category to the test species of interest.

The physical characteristics scored include color, polyp extension/retraction, tissue swelling/distension, tissue attenuation, and mucus production. These observable, physical characteristics are not independent, as changes to one characteristic may or may not reflect alterations in another. Each of the five characteristics are scored on a scale of 0 (normal limits) to 3 (severely affected), with a precision level of 0.5 (thus half-scores are permitted) (Supplemental Table 1). The maximum score for an individual coral is 12; tissue swelling and tissue attenuation are scored separately but are considered as one category, as the two criteria are opposite responses to stress (a coral which receives an attenuation score of 3 cannot have a swelling score greater than 0, as there is no tissue to swell). To determine the EC50, the scores for each criterion were summed and divided by the total maximum score possible (12) to obtain a single percent effect for each coral fragment at each time point.

Color: Scores for changes (loss) in coral color were used to assess relative lightening (or bleaching) of coral fragments. Due to inherent variation in coral fragment color at the beginning of each exposure, this score was based on the lightening of color of each fragment relative to the fragment's initial color. A score of 0 was given to a coral which has maintained its coloration, and a coral with slight lightening on color received a score of 1. A score of 2 represents a coral that is moderately bleached, and a score of 3 indicated significant bleaching/loss of color. To score color correctly, the corals must be evaluated and photographed under the same lighting conditions at each time point, and photos taken before and after the exposure for verification must be consistently white balanced.

Polyps: Polyps which were normally extended or slightly retracted received a score of 0. Polyps which were retracted and partly closed received a score of 1; this score would be representative of a "startled" polyp. *Great care must be taken during scoring to avoid disturbing the corals and therefore causing a polyp score not related to exposure conditions.* Fully closed polyps received a score of 2, and those with very tightly retracted polyps received a score of 3. It is important to

note that the degree of polyp retraction associated with scores of 2-3 was not readily reversible; the polyps were completely contracted/recessed and no tentacular tissue was visible. This extreme retraction represented a significant disruption in normal coral behavior, and frequently included exposure of skeletal elements around the polyp; corals with high polyp scores often had very little tissue outside of the corallites. Thus, severe polyp retraction could be accompanied by tissue attenuation.

Supplemental Table S1. Coral condition categories and description of score qualifications.

Diagnostic Criteria	Range
Color	• 0 (normal): color appears normal<br>• 1 (mild): slight lightening of coloration<br>• 2 (moderate): moderate lightening of coloration<br>• 3 (severe): significant lightening of coloration, evident bleaching
Polyps	• 0 (normal): fully extended or loosely retracted<br>• 1 (mild): retracted and slightly closed<br>• 2 (moderate): evident polyp retraction with full polyp closure<br>• 3 (severe): polyps tightly retracted
Tissue swelling	• 0 (normal): no swelling<br>• 1 (mild): slight coenenchyme swelling and/or polyp distension<br>• 2 (moderate): moderate coenenchyme swelling and/or polyp distension<br>• 3 (severe): severe swelling of coenenchyme and/or polyp distension
Tissue attenuation	• 0 (normal): no attenuation<br>• 1 (mild): slight thinning of coenenchyme, flattening of polyps<br>• 2 (moderate): moderate thinning of coenenchyme and polyp flattening<br>• 3 (severe): severe tissue thinning, skeletal ridges exposed
Mucus production	• 0 (normal): normal mucus production; no mesenterial filaments apparent<br>• 1 (mild): slightly elevated mucus production, no mesenterial filaments apparent<br>• 2 (moderate): moderately elevated mucus production; mesenterial filament extrusion possible<br>• 3 (severe): mucus sheets evident; possible mesenterial filament extrusion

Tissue swelling: A tissue swelling score of 1 indicated a slight localized or diffuse swelling, usually of the coenenchyme. More extensive coenenchyme swelling and/or a greater degree of polyp distension received a score of 2, and extreme swelling of coral tissues and/or extreme polyp distension received a score of 3. Scores of 2-3 often indicated variable severity of diffuse swelling throughout the coenenchyme of the entire coral. In some cases, severe swelling manifested as large bubbles under localized areas of tissue, engulfing the coenenchyme and polyp. These "bubbles" frequently resulted in eventual tissue lysis of the affected area.

Tissue attenuation: A minor thinning of the coenenchyme, and/or slight recession of tissue from corallite ridges resulted in a score of 1. A greater degree of thinning, typically accompanied by clear visualization (but not exposure) of skeletal elements received a score of 2. Severe tissue thinning was characterized by exposure of skeletal elements in the coenenchyme and/or the polyp (sclerosepta and columella exposed within polyps) and was given a score of 3.

Mucus production: All corals continually produce some amount of mucus; an amount produced somewhat greater than this level received a score of 1. Low mucus scores were indicative of a localized mucus release, or a thin halo that was observable with backlighting. A score of 2 was given to corals producing more significant amounts of mucus, with more visible mucus strings or thin sheets extending upward from the coral. Partial mesenterial filament extrusion was possible but not frequently observed. Corals producing copious amounts of mucus, in thick sheets or with pools of mucus around the base of the coral received a score of 3. Again, mesenterial filament extrusion was possible but not typically seen in these exposures.

Mucus production was observed to have a high temporal variability, depending on the toxicant concentration. Low concentrations usually resulted in slower, consistently elevated mucus secretion that occurred over the duration of the exposure. High concentrations resulted in copious mucus production early in the exposure which then decreased later in the exposure, likely related to a depletion of energetic resources used to support mucus production. Mucus secretion was therefore not a reversible/irreversible stress, but rather a time/concentration dependent depletion of organism resources. For this reason, mucus scores that reached a high- to severely-impacted level (2.5-3) were fixed at subsequent time points, even if mucus production subsequently decreased as a result of tissue degradation or necrosis.

Supplemental Table S2. Diving-PAM light-adapted effective quantum yield determination parameters used for each coral species.

Organism	Measuring light intensity	Damping	Gain	Saturation Intensity	Saturation Width
Acropora cervicornis	5	2	4	8	0.8
Porites astreoides	3	2	3	8	0.8
Siderastrea siderea	3	2	4	8	0.8
Solenastrea bournoni	3	2	3	8	0.8
Stephanocoenia intersepta	3	2	3	8	0.8

Supplemental Table S3. Mean (±SD) water quality parameters for each exposure.

Coral	Treatment (µg/L 1-MN)	ºC	$pH_{(NBS)}$	DO (mg/L)	Alk (mg/L $CaCO_3$)	PO_4 (mg/L PO_4)	NH_3 (mg/L NH_3-N)	NO_2 (mg/L NO_2-N)	NO_3 (mg/L NO_3-N)
Acropora cervicornis	Control	26.8(±0.17)	7.89(±0.08)	7.7(±0.34)	108.1(±6.1)	0.31(±0.23)	0.18(±0.13)	0.013(±0.003)	0.03(±0.01)
	745	26.9(±0.08)	7.87(±0.06)	6.9(±0.10)	112.8(±4.7)	0.14(±0.09)	0.00(±0.00)	0.013(±0.001)	0.03(±0.01)
	1501	26.7(±0.28)	7.93(±0.05)	7.1(±0.33)	115.8(±5.9)	0.26(±0.17)	0.02(±0.05)	0.019(±0.001)	0.03(±0.01)
	2775	26.8(±0.22)	7.80(±0.04)	6.7(±0.36)	113.3(±7.1)	0.15(±0.06)	0.01(±0.01)	0.022(±0.009)	0.03(±0.01)
	5370	26.8(±0.14)	7.06(±0.13)	3.6(±0.34)	119.6(±7.3)	0.42(±0.15)	0.00(±0.00)	0.033(±0.006)	0.08(±0.02)
	9434	26.8(±0.25)	6.75(±0.04)	2.2(±0.17)	125.0(±2.8)	0.48(±0.13)	0.09(±0.09)	0.038(±0.007)	0.10(±0.02)
Porites astreoides	Control	26.2(±0.21)	8.18(±0.02)	5.0(±0.01)	132.4(±0.8)	0.20(±0.08)	0.00(±0.00)	0.010(±0.002)	0.02(±0.01)
	1522	26.2(±0.18)	8.18(±0.01)	4.8(±0.15)	134.4(±1.3)	0.22(±0.05)	0.00(±0.00)	0.011(±0.002)	0.02(±0.01)
	2868	26.1(±0.15)	8.18(±0.01)	4.7(±0.13)	134.9(±0.9)	0.25(±0.07)	0.00(±0.00)	0.011(±0.005)	0.02(±0.01)
	5236	26.2(±0.17)	8.19(±0.01)	4.3(±0.24)	136.9(±1.4)	0.22(±0.04)	0.00(±0.00)	0.011(±0.001)	0.02(±0.01)
	8293	26.2(±0.21)	8.16(±0.01)	4.4(±0.25)	138.8(±0.6)	0.21(±0.06)	0.03(±0.05)	0.012(±0.003)	0.03(±0.01)
	12530	26.2(±0.24)	8.13(±0.04)	3.6(±0.26)	141.1(±0.4)	0.28(±0.04)	0.04(±0.04)	0.016(±0.005)	0.03(±0.01)
Siderastrea siderea	Control	22.2(±0.17)	7.78(±0.05)	*9.5(±0.42)	108.5(±3.3)	0.22(±0.10)	0.01(±0.01)	0.009(±0.002)	0.05(±0.01)
	828	22.3(±0.24)	7.77(±0.05)	*9.4(±0.24)	113.1(±3.0)	0.35(±0.13)	0.01(±0.03)	0.008(±0.001)	0.05(±0.01)
	1614	21.9(±0.58)	7.71(±0.07)	*8.8(±0.13)	112.8(±0.6)	0.24(±0.09)	0.02(±0.02)	0.008(±0.004)	0.06(±0.01)
	3030	22.2(±0.39)	7.79(±0.02)	*8.7(±0.17)	115.2(±3.6)	0.23(±0.15)	0.02(±0.03)	0.009(±0.001)	0.07(±0.01)
	5876	22.2(±0.24)	7.79(±0.02)	*8.4(±0.24)	121.7(±2.7)	0.38(±0.12)	0.02(±0.02)	0.009(±0.001)	0.07(±0.01)
	10332	22.1(±0.54)	7.70(±0.03)	*7.6(±0.34)	125.8(±3.5)	0.27(±0.20)	0.03(±0.03)	0.008(±0.003)	0.08(±0.01)
Stephanocoenia intersepta	Control	25.2(±0.21)	7.99(±0.01)	7.9(±0.21)	115.6(±8.8)	0.18(±0.06)	0.02(±0.02)	0.016(±0.003)	0.05(±0.01)
	805	25.3(±0.21)	7.89(±0.05)	7.2(±0.39)	97.1(±7.4)	0.17(±0.07)	0.01(±0.01)	0.017(±0.007)	0.04(±0.01)
	1616	25.2(±0.26)	7.91(±0.02)	7.4(±0.30)	98.0(±2.8)	0.29(±0.31)	0.04(±0.03)	0.018(±0.005)	0.05(±0.02)
	2955	25.4(±0.33)	7.85(±0.06)	6.9(±0.34)	99.1(±6.5)	0.40(±0.65)	0.01(±0.01)	0.015(±0.006)	0.05(±0.01)
	5610	25.3(±0.29)	7.85(±0.03)	6.9(±0.20)	104.9(±6.3)	0.14(±0.08)	0.00(±0.00)	0.016(±0.005)	0.05(±0.01)
	9019	25.3(±0.26)	7.81(±0.04)	6.6(±0.13)	99.5(±1.7)	0.80(±1.30)	0.03(±0.07)	0.014(±0.006)	0.04(±0.02)
Solenastrea bournoni	Control	25.1(±0.22)	8.05(±0.04)	7.7(±0.29)	123.6(±2.0)	0.12(±0.07)	0.09(±0.12)	0.015(±0.012)	0.07(±0.01)
	788	25.1(±0.15)	8.02(±0.03)	7.1(±0.10)	122.4(±2.6)	0.15(±0.13)	0.01(±0.01)	0.005(±0.002)	0.07(±0.01)
	1719	25.1(±0.29)	8.01(±0.01)	7.1(±0.12)	124.7(±4.3)	0.18(±0.21)	0.00(±0.01)	0.009(±0.005)	0.06(±0.01)
	3081	25.2(±0.32)	7.97(±0.05)	6.8(±0.05)	123.5(±1.6)	0.23(±0.31)	0.05(±0.06)	0.008(±0.006)	0.06(±0.01)
	5712	25.1(±0.29)	7.96(±0.04)	6.5(±0.22)	125.2(±2.1)	0.44(±0.24)	0.00(±0.00)	0.010(±0.007)	0.07(±0.02)
	10293	25.1(±0.46)	7.87(±0.04)	6.4(±0.16)	123.5(±2.2)	0.25(±0.38)	0.02(±0.04)	0.010(±0.007)	0.07(±0.01)

*abnormally high values attributed to instrument miscalibration.

Supplemental Table S4. Kruskal-Wallis ANOVA results for coral condition statistical comparisons.

Coral	Time	Kruskal-Wallis H	*p* value	Time	Kruskal-Wallis H	*p* value
Acropora cervicornis	1 h (Exp)	$H_{(5,24)}$ = 19.15855	0.0018	8 h (Exp)	$H_{(5,24)}$ = 19.44949	0.0016
	2 h (Exp)	$H_{(5,24)}$ = 21.03971	0.0008	12 h (Exp)	$H_{(5,24)}$ = 18.75055	0.0021
	3 h (Exp)	$H_{(5,24)}$ = 20.27417	0.0011	24 h (Exp)	$H_{(5,24)}$ = 20.07972	0.0012
	4 h (Exp)	$H_{(5,24)}$ = 20.21901	0.0011	36 h (Exp)	$H_{(5,24)}$ = 20.69374	0.0009
	5 h (Exp)	$H_{(5,24)}$ = 19.04103	0.0019	48 h (Exp)	$H_{(5,24)}$ = 19.03941	0.0019
	6 h (Exp)	$H_{(5,24)}$ = 19.07768	0.0019	1 wk (Post)	$H_{(3,16)}$ = 2.836538	0.4175
	7 h (Exp)	$H_{(5,24)}$ = 20.25456	0.0011	4 wk (Post)	$H_{(3,8)}$ = 00.00000	1.0000
Porites astreoides	1 h (Exp)	$H_{(5,24)}$ = 18.00270	0.0029	8 h (Exp)	$H_{(5,24)}$ = 21.70437	0.0006
	2 h (Exp)	$H_{(5,24)}$ = 18.00270	0.0029	12 h (Exp)	$H_{(5,24)}$ = 21.86707	0.0006
	3 h (Exp)	$H_{(5,24)}$ = 17.05500	0.0044	24 h (Exp)	$H_{(5,24)}$ = 20.96852	0.0008
	4 h (Exp)	$H_{(5,24)}$ = 20.52438	0.0010	36 h (Exp)	$H_{(5,24)}$ = 19.91003	0.0013
	5 h (Exp)	$H_{(5,24)}$ = 21.70852	0.0006	48 h (Exp)	$H_{(5,24)}$ = 19.88943	0.0013
	6 h (Exp)	$H_{(5,24)}$ = 21.37833	0.0007	1 wk (Post)	$H_{(5,24)}$ = 20.02550	0.0012
	7 h (Exp)	$H_{(5,24)}$ = 21.32764	0.0007	4 wk (Post)	$H_{(5,11)}$ = 6.671271	0.2463
Siderastrea siderea	1 h (Exp)	$H_{(5,24)}$ = 15.16531	0.0097	8 h (Exp)	$H_{(5,24)}$ = 21.01354	0.0008
	2 h (Exp)	$H_{(5,24)}$ = 18.93821	0.0020	12 h (Exp)	$H_{(5,24)}$ = 19.15912	0.0018
	3 h (Exp)	$H_{(5,24)}$ = 16.62200	0.0053	24 h (Exp)	$H_{(5,24)}$ = 20.76291	0.0009
	4 h (Exp)	$H_{(5,24)}$ = 11.96061	0.0353	36 h (Exp)	$H_{(5,24)}$ = 20.70851	0.0009
	5 h (Exp)	$H_{(5,24)}$ = 15.91244	0.0071	48 h (Exp)	$H_{(5,24)}$ = 22.29847	0.0005
	6 h (Exp)	$H_{(5,24)}$ = 13.94261	0.0160	1 wk (Post)	$H_{(5,24)}$ = 20.08400	0.0012
	7 h (Exp)	$H_{(5,24)}$ = 18.21715	0.0027	4 wk (Post)	$H_{(5,11)}$ = 9.308252	0.0974
Solenastrea bournoni	1 h (Exp)	$H_{(5,24)}$ = 20.97604	0.0008	8 h (Exp)	$H_{(5,24)}$ = 18.49671	0.0024
	2 h (Exp)	$H_{(5,24)}$ = 16.67475	0.0052	12 h (Exp)	$H_{(5,24)}$ = 19.57756	0.0015
	3 h (Exp)	$H_{(5,24)}$ = 19.84900	0.0013	24 h (Exp)	$H_{(5,24)}$ = 20.32371	0.0011
	4 h (Exp)	$H_{(5,24)}$ = 19.37362	0.0016	36 h (Exp)	$H_{(5,24)}$ = 20.78711	0.0009
	5 h (Exp)	$H_{(5,24)}$ = 20.66281	0.0009	48 h (Exp)	$H_{(5,24)}$ = 21.49135	0.0007
	6 h (Exp)	$H_{(5,24)}$ = 19.62697	0.0015	1 wk (Post)	$H_{(5,24)}$ = 21.49818	0.0007
	7 h (Exp)	$H_{(5,24)}$ = 20.34210	0.0011	4 wk (Post)	$H_{(5,12)}$ = 7.526316	0.1843
Stephanocoenia intersepta	1 h (Exp)	$H_{(5,24)}$ = 20.23138	0.0011	8 h (Exp)	$H_{(5,24)}$ = 4.234694	0.561
	2 h (Exp)	$H_{(5,24)}$ = 20.26875	0.0011	12 h (Exp)	$H_{(5,24)}$ = 9.430000	0.0931
	3 h (Exp)	$H_{(5,24)}$ = 15.30209	0.0091	24 h (Exp)	$H_{(5,24)}$ = 15.08806	0.0100
	4 h (Exp)	$H_{(5,24)}$ = 17.78395	0.0032	36 h (Exp)	$H_{(5,24)}$ = 12.85725	0.0248
	5 h (Exp)	$H_{(5,24)}$ = 11.85443	0.0368	48 h (Exp)	$H_{(5,24)}$ = 12.85725	0.0248
	6 h (Exp)	$H_{(5,24)}$ = 11.03724	0.0506	1 wk (Post)	$H_{(5,24)}$ = 6.337461	0.2748
	7 h (Exp)	$H_{(5,24)}$ = 9.327494	0.0967	4 wk (Post)	$H_{(5,12)}$ = 00.00000	1.0000

Supplemental Table S5. One-way ANOVA results for photosynthetic efficiency statistical comparisons.

Coral	Time	DF	SS	MS	F value	*p* value
Acropora cervicornis	Pre-exposure (1 wk)	5	0.000265	0.000053	0.535747	0.746627
	Exposure (48 h)	3	0.005455	0.001818	29.19974	0.000008
	Post-Exposure (1 wk)	3	0.000749	0.000250	1.084915	0.392642
	Post-Exposure (4 wk)	Kruskal Wallis ANOVA, $H_{(3,8)}$ = 2.833333, p=0.4180				
Porites astreoides	Pre-exposure (1 wk)	5	0.000641	0.000128	1.816771	0.160255
	Exposure (48 h)	5	0.060455	0.012091	3.384772	0.024923
	Post-Exposure (1 wk)	5	0.019617	0.003923	2.153237	0.105383
	Post-Exposure (1 wk)	Kruskal Wallis ANOVA, $H_{(5,11)}$ = 6.090909, p=0.2975				
Siderastrea siderea	Pre-exposure (1 wk)	5	0.000153	0.000031	0.562869	0.727157
	Exposure (48 h)	5	0.227831	0.045566	24.039458	0.000000
	Post-Exposure (1 wk)	5	0.007551	0.001510	0.897445	0.503763
	Post-Exposure (4 wk)	Kruskal Wallis ANOVA, $H_{(5,11)}$ = 6.090909, p=0.2975				
Solenastrea bournoni	Pre-exposure (1 wk)	5	0.000339	0.000068	0.952851	0.471691
	Exposure (48 h)	5	0.067786	0.013557	45.033312	0.000000
	Post-Exposure (1 wk)	5	0.011682	0.002336	9.642805	0.000131
	Post-Exposure (1 wk)	Kruskal Wallis ANOVA, $H_{(5,12)}$ = 4.564103 p=0.4714				
Stephanocoenia intersepta	Pre-exposure (1 wk)	5	0.000215	0.000043	0.426358	0.824294
	Exposure (48 h)	5	0.152438	0.030488	90.297088	0.000000
	Post-Exposure (1 wk)	5	0.001258	0.000252	0.937165	0.480606
	Post-Exposure (4 wk)	Kruskal Wallis ANOVA, $H_{(5,12)}$ = 2.923077 p=0.7118				

Supplemental Table S6. Kruskal-Wallis ANOVA results for coral mortality statistical comparisons.

Coral	Time	Kruskal-Wallis H	*p* value
Acropora cervicornis	24 h (Exp)	$H_{(5,24)}$ = 19.50038	0.0016
	36 h (Exp)	$H_{(5,24)}$ = 22.64706	0.0004
	48 h (Exp)	$H_{(5,24)}$ = 20.48254	0.0010
	1 wk (Post)	$H_{(3,16)}$ = 00.00000	1.0000
	4 wk (Post)	$H_{(3,8)}$ = 00.00000	1.0000
Porites astreoides	24 h (Exp)	$H_{(5,24)}$ = 17.00173	0.0045
	36 h (Exp)	$H_{(5,24)}$ = 17.64342	0.0034
	48 h (Exp)	$H_{(5,24)}$ = 21.46667	0.0007
	1 wk (Post)	$H_{(5,24)}$ = 5.992287	0.3070
	4 wk (Post)	$H_{(5,11)}$ = 0.000000	1.0000
Siderastrea siderea	24 h (Exp)	$H_{(5,24)}$ = 00.00000	1.0000
	36 h (Exp)	$H_{(5,24)}$ = 00.00000	1.0000
	48 h (Exp)	$H_{(5,24)}$ = 19.84469	0.0013
	1 wk (Post)	$H_{(5,24)}$ = 18.84500	0.0021
	4 wk (Post)	$H_{(5,11)}$ = 4.500000	0.4799
Solenastrea bournoni	24 h (Exp)	$H_{(5,24)}$ = 00.00000	1.0000
	36 h (Exp)	$H_{(5,24)}$ = 00.00000	1.0000
	48 h (Exp)	$H_{(5,24)}$ = 17.64193	0.0034
	1 wk (Post)	$H_{(5,24)}$ = 5.000000	0.4159
	4 wk (Post)	$H_{(5,12)}$ = 5.000000	0.4159
Stephanocoenia intersepta	24 h (Exp)	$H_{(5,24)}$ = 10.45455	0.0633
	36 h (Exp)	$H_{(5,24)}$ = 10.45455	0.0633
	48 h (Exp)	$H_{(5,24)}$ = 22.85714	0.0004
	1 wk (Post)	$H_{(5,24)}$ = 4.184783	0.5231
	4 wk (Post)	$H_{(5,12)}$ = 00.00000	1.0000

Supplemental Table S7. Subacute and acute endpoints [in µg/L 1-MN (95% CI)] determined at each time point for coral species exposed to 1-methylnaphthalene.

Coral	Time (h)	$EC10_{Condition}$ (µg/L 1-MN)	$EC50_{Condition}$ (µg/L 1-MN)	LC50 (µg/L 1-MN)
Acropora cervicornis	12 (Exp)	-	13890*	-
	24 (Exp)	2366 (1258-3474)	4409 (3568-5249)	5524 (5378-5671)
	36 (Exp)	2560 (2004-3116)	3476 (2998-3953)	4571 (3022-6120)
	48 (Exp)	1943 (1013-2872)	3126 (2574-3678)	3421 (2670-4174)
Porites astreoides	12 (Exp)	4093 (2908-5279)	9330 (8414-10246)	-
	24 (Exp)	4109 (3230-4989)	7306 (6678-7935)	-
	36 (Exp)	4305 (3426-5185)	6775 (6245-7305)	-
	48 (Exp)	4593 (4342-4844)	5819 (5594-6045)	11982 (9017-14949)
Siderastrea siderea	12 (Exp)	2312 (0-6271)	20723*	-
	24 (Exp)	862 (0-2253)	8148 (0-38716)	-
	36 (Exp)	1379 (719-2039)	7357 (6136-8579)	-
	48 (Exp)	857 (596-1118)	5189 (4583-5794)	> solubility
Solenastrea bournoni	12 (Exp)	1493 (287-2699)		
	24 (Exp)			
	36 (Exp)	861 (17-1705)		
	48 (Exp)	2355 (1048-3663)	7127 (5945-8309)	> solubility
Stephanocoenia intersepta	12 (Exp)	4351	-	-
	24 (Exp)	615	-	-
	36 (Exp)	-	-	-
	48 (Exp)	673 (42-1305)	9294 (6370-12217)	11787 (4956-18618)

*above highest measured 1-MN concentration
